# Cumulative adulthood trauma exposures and pain outcomes among older adults

**DOI:** 10.1177/20494637261471088

**Published:** 2026-07-22

**Authors:** Yeonwoo Kim, Lisa S. Panisch, Soeun Jang

**Affiliations:** 1 Department of Kinesiology, 12329 University of Texas at Arlington, Arlington, TX, USA; 2 School of Social Work, 2954 Wayne State University, Detroit, MI, USA; 3 School of Social Work, 12329 University of Texas at Arlington, Arlington, TX, USA

**Keywords:** middle-aged and older adults, adulthood trauma, pain onset, pain prevalence, cumulative trauma

## Abstract

**Objective:**

The majority of research on connections between trauma exposure and adult pain focuses on childhood trauma. Trauma experienced during adulthood receives less attention, especially among middle-aged and older adults. This longitudinal study explores the relationships between adulthood trauma and the onset of pain over 6 years among middle-aged and older adults, after controlling for childhood trauma.

**Method:**

We analyze data from the Health and Retirement Study, a nationally representative longitudinal survey of adults aged 50+. Our analytic sample (*N* = 6724) consists of participants (*M* = 64.4 ± 10.8 years) with no reports of pain in the past 10 years. A multilevel mixed effects logistic regression analysis was performed with repeated measures of pain over a six-year period as an outcome, adulthood traumatic events as exposures, and demographic, childhood trauma, and health-related factors as covariates.

**Results:**

Exposure to 2+ adulthood traumatic events was significantly related to a higher risk of pain onset (OR = 1.34; 95% CI: 1.04, 1.71), when controlling for demographic and health-related factors. After adjusting for childhood trauma, this relationship attenuated slightly, but remained statistically significant (OR = 1.28; 95% CI: 1.00, 1.64).

**Conclusions and implications:**

Our results indicate that trauma exposure at multiple life stages independently contributes to pain outcomes in older adults. Notably, the negative impact of adulthood trauma persists even among those with a history of childhood trauma. We encourage future research exploring these underlying biopsychosocial mechanisms linking life-course trauma to pain in later life.

## Introduction

Pain experienced in older adulthood is a multifaceted health concern. The prevalence of chronic pain among older adults ranges from approximately 27–52%, substantially higher than the 20% prevalence observed in the general adult population.^1–3^ Chronic, or persistent, pain refers to a persistent experience of aversive sensory and emotional stimuli that is not dependent upon the presence of tissue injury or related pain-inducing phenomena.^4,5^ Late-life chronic pain is linked to poor mental health, increased suicide risk, and reduced quality of life and is more complex and difficult to manage compared to younger populations.^6,7^

Chronic pain can be exacerbated by exposures to traumatic experiences throughout one’s life.^5,7^ There is a growing body of research examining the effect of childhood traumatic events on pain in middle-aged and older adulthood. Negative effects of childhood trauma may impact youth’s brain and nervous system development in a manner that yields life-long consequences, including greater risk of major illness, poor mental health outcomes, and chronic pain.^8,9^ The long-term consequences of cumulative childhood trauma exposures, such as different types of abuse and household dysfunction, are known to have a negative impact on adult health in a dose-response manner, meaning that greater traumatic exposure is associated with higher risk for adverse adult health outcomes.^9–11^ Importantly, researchers have shown that assessing the cumulative frequency and variety of traumatic experiences provides a more robust prediction of pain outcomes than simply examining the presence or absence of a single trauma type.^11^

Less attention has been given to the independent contributions of adult trauma exposures to pain outcomes after controlling for childhood trauma, especially among older adults.^5^ To our knowledge, the few existing studies on this topic have all relied on cross-sectional data. Among these limited cross-sectional studies, findings remain inconsistent regarding the relationships between adulthood versus childhood exposure to trauma and adult pain outcomes in later life.^12–14^ A study in South Africa showed higher odds of late-life chronic pain in relationship to adult trauma exposures like violence exposure, illness, accidents, and disasters, while no significant associations were found between childhood trauma exposures (household dysfunction and physical abuse) and late-life pain.^14^ In contrast, another study^13^ suggested that childhood trauma may have a stronger impact on pain outcomes (i.e., debilitating chronic pain that limits one’s functioning) than adulthood trauma by reporting lower prevalence rates of pain among women exposed to trauma in adulthood only, in comparison to women who had only been exposed to trauma in childhood, as well as those with childhood and adulthood trauma exposures. Additionally, they found greater pain prevalence among people exposed to both childhood and adult trauma,^13^ as well as a dose-response relationship between greater lifetime cumulative exposures to trauma and higher risk of more severe pain.^14^ Another study comparing childhood vs adulthood exposure to trauma, specifically abuse, did not find differences in pain outcomes based on the age of exposure.^12^ Rather, lifetime exposure to abuse at any point was associated with higher pain severity and lower pain interference, as well as more impaired physical functioning and worse mental health outcomes. Other studies examining links between trauma exposure and pain in older adults have collapsed variables distinguishing between childhood and adult trauma exposure into a single lifetime exposure variable, making it difficult to distinguish the independent contributions of each.^15^ These findings suggest a need for more research that isolates adulthood trauma exposure and considers its cumulative impact on later-life pain outcomes.

The importance of accounting for childhood trauma when examining the impact of adulthood trauma on pain is further underscored by evidence showing that childhood and adulthood trauma exposures are often interrelated. For example, researchers have demonstrated higher rates of childhood trauma exposures among adults who go on to develop PTSD after adult exposure to trauma in combat.^16^ Similar results have been reported by researchers studying connections between childhood trauma exposure and subsequent adulthood exposure to domestic and intimate partner violence.^17^ Findings of this nature indicate that childhood traumatic exposures may increase vulnerability to adulthood exposures,^17^ further underscoring the need to distinguish between childhood and adulthood trauma exposures when studying relationships between trauma and pain outcomes in older adults.

Some of the existing literature examines common covariates of chronic pain among older adults, including comorbid chronic physical and mental health conditions, as well as ratings of their own health.^18,19^ For example, both pain- and non-pain-related comorbid physical health conditions were associated with low back pain among older adults.^19^ Chronic pain risk was greater among older adults with more physical health comorbidities^7,19^; this relationship persisted even when only non-pain-related conditions were examined.^18^ Pain was also more commonly reported among older adults with poor self-ratings of health,^19^ and higher levels of pain severity were also associated with fair or poor self-rated health among US adults in general.^20^ Mental health conditions, particularly depression, are commonly experienced by older adults with chronic pain and have been associated with higher pain prevalence, severity, and intensity in a potentially bi-directional relationship.^21,22^ Both depression and pain are commonly reported among older adults exposed to trauma at any age.^7,23^

### Study purpose

Prior research offers mixed results on whether traumatic exposure in adulthood has a unique contribution to pain in middle-aged and older adults, and all of this research is based on cross-sectional data.^12–14^ While numerous studies have examined both specific and cumulative childhood trauma,^8–11^ fewer have explored the cumulative effects of adulthood trauma across different types of exposures.^13,15,24^ Therefore, the long-term impact of cumulative adulthood-onset trauma on pain outcomes remains unclear. This longitudinal study addresses that gap by focusing on middle-aged and older adults who had not reported moderate-to-severe pain in the 10 years prior to baseline, and by examining the relationships between cumulative adulthood trauma exposure and later-life pain outcomes over 6 years, after controlling for childhood trauma exposure. In this study, adulthood trauma exposure is defined as one or more adverse experiences, including but not limited to having lost a child, having fired a weapon in combat or been fired upon in combat, or having had a spouse or child who experienced a life-threatening illness or accident. Childhood trauma exposure is defined as one or more adverse experiences including repeating a year of school, having trouble with the police, parental alcohol or drug use that caused family problems, and physical abuse by a parent. We hypothesize the following: (1) Middle-aged and Older adults with a history of adulthood trauma exposure are more likely to report pain over 6 years than those who did not experience a traumatic event; (2) Cumulative adulthood trauma exposures will be associated with higher pain prevalence among middle-aged and older adults, and (3) Middle-aged and Older adults who experienced cumulative trauma in both childhood and adulthood are more likely to report greater pain prevalence than adults exposed to trauma in childhood or adulthood only.

## Methods

### Dataset

This study obtained data from the Health and Retirement Study (HRS), an ongoing, nationally representative study of Americans aged 50 and older.^25^ The HRS began recruiting in 1992-1993 with additional birth cohorts added in 1998, 2004, 2010, and 2016, and has collected data biennially from participants and their spouses. Since 2016, the HRS has implemented the Leave Behind Survey, which included questions about traumatic events. After finishing the HRS core survey, respondents answered the Leave Behind Survey every four years, as it was conducted for a random half of the participants for each biennial wave.

For this study, we analyzed data from the HRS 2012–2018. We only included respondents aged 50+ years old who were free of moderate-to-severe pain from 2002 to 2012 (*N* = 7333). We excluded missing data on traumatic events (*n* = 379), race/ethnicity (*n* = 46), education (*n* = 8), and health status (*n* = 123). To avoid reverse causality, we excluded those who reported adulthood traumatic events from 2014 onward, when the outcome measure was first assessed (*n* = 53), resulting in 6724 respondents.

### Measures

#### Onset of moderate-to-severe pain

Our outcome measure was the onset of moderate-to-severe pain from 2014 to 2018. Pain was assessed using a three-step series of questions based on previous literature.^24,26,27^ First, respondents were asked: “Are you often in trouble with pain?” (yes or no). Respondents who answered “yes” were classified as experiencing chronic pain. Two follow-up questions were then asked to those with chronic pain: (1) “How bad is the pain most of the time?” (mild, moderate, or severe) and (2) “Does the pain make it difficult for you to do your usual activities such as household chores or work?” (yes or no). Respondents who reported moderate or severe pain in the second question or answered “yes” to the third question were classified as experiencing moderate-to-severe pain (=1). All others, including those with no chronic pain or only mild pain, were classified as no moderate-to-severe pain (=0).

#### Adulthood traumatic events

Adulthood traumatic events were assessed as the exposure variables, using items from the HRS Psychosocial and Lifestyle Questionnaire, adapted from the work of Krause.^28^ This measurement is rooted in previous literature^29,30^ and used in many studies of traumatic events and health.^31–33^ Respondents answered seven questions about whether they had experienced any of the following events at any point in their lives: (1) having lost a child, (2) having experienced a major fire, flood, earthquake, or natural disaster, (3) having fired a weapon in combat or been fired upon in combat, (4) having had a spouse, partner, or child addicted to drugs or alcohol, (5) having been the victim of a serious physical attack or assault, (6) having had a life-threatening illness or accident, and (7) having had a spouse or child who experienced a life-threatening illness or accident. If participants reported experiencing, they were asked for the year of the most recent occurrence. Events that occurred before age 18 were excluded from the adulthood traumatic event count. Then, we calculated a cumulative score of adulthood traumatic events by summing the number of different adulthood traumatic events experienced (range: 0–7).

Following previous literature,^28,34^ a cumulative score of adulthood traumatic events was calculated by summing the number of different adulthood traumatic events experienced (range: 0–7). To examine the non-linear relationship of adulthood traumatic events with the onset of pain, we categorized the score into three categories: (1) no event, (2) one event, and (3) two events or more.

#### Childhood traumatic events

Childhood traumatic events were assessed using a subset of items from the HRS Psychosocial and Lifestyle Questionnaire traumatic events checklist, adapted from the work of Krause.^28^ This subset was restricted to events reported to have occurred prior to age 18 and has been used in previous studies assessing childhood traumatic events.^35,36^ Four items included: repeating a year of school, having had trouble with the police, parental alcohol or drug use that caused family problems, and physical abuse by a parent. We calculated a summary score by summing the number of different events (range: 0–4). To assess the non-linearity of childhood traumatic events with the onset of pain, we categorized the score into three categories: (1) no event, (2) one event, and (3) two events or more.

### Sociodemographic and health-related characteristics

Sociodemographic characteristics were included as covariates. Respondents’ age, gender, race/ethnicity, and education level at baseline were considered as time-invariant covariates. Annual household income was assessed as time-variant to account for changes in financial stability over time. Additionally, to address the effect of physical and mental health on the onset of pain, we included time-variant self-rated physical health based on the questionnaire asking to rate their health on a 5-point scale ranging from 1 (excellent) to 5 (poor) and dichotomized into good health (excellent [1], very good [2], or good [3] = 1) and poor health (fair [4] or poor [5] = 0). Additionally, the number of chronic diseases, including arthritis, cancer, diabetes, heart disease, high blood pressure, lung disease, and stroke, was included as a covariate. Depressive symptoms, measured using the Center for Epidemiologic Studies Depression (CES-D) scale,^37^ were also included as an additional covariate.

### Statistical analysis

Descriptive and bivariate analyses were conducted to examine the sample characteristics of the respondents and the differences in the onset of pain across experiences of childhood and adulthood traumatic events. A multilevel mixed effects logistic regression analysis was performed with repeated measures of pain (*t*) as an outcome measure and adulthood traumatic events as the exposures. Annual household income, physical health, and mental health (*t*-1) were included as time-variant covariates, while childhood traumatic events, age, gender, race/ethnicity, and education at baseline were included as time-invariant covariates. We examined the time-lagged effect by modeling exposure and time-variant covariates from the prior wave (*t*-1) to assess their associations with pain onset at the current wave (*t*). Initially, we included adulthood traumatic events in the model, along with sociodemographic characteristics. We also tested its influence on the trajectories of moderate-to-severe pain over time by including time interaction terms (i.e., adulthood traumatic events by time interaction) and removing nonsignificant interaction terms. Second, we added time-variant physical and mental health measures as covariates in the model to examine whether the association between adulthood traumatic events and pain changed when adjusting for health-related covariates. Finally, we additionally adjusted for childhood traumatic events in the model. During the model fitting process, we evaluated best-fitting models based on the Akaike Information Criterion, Bayesian Information Criterion, coefficient significance, and model parsimony. We used survey procedures to account for the complex survey design of the HRS. Analyses were performed using Stata Version 16.

## Results

Table 1 presents the demographic characteristics of the sample at baseline. The average age was 67 years (*SD* = 10.4; range: 50–100), and half of the sample was female (55%). The majority self-identified as non-Hispanic White (69%), and 63% were married. One-eighth rated their health as poor (12%), and half had no chronic conditions at baseline (59%). Their average depression score was 0.8 (*SD* = 1.4; range: 0–8). Regarding childhood traumatic events, 30% of the respondents reported experiencing childhood traumatic events, with 23% reporting one event and 7% reporting two or more events. Adulthood traumatic events were reported by 54% of the respondents, with 30% reporting one, 25% reporting two or more events.

**Table 1.** Descriptive characteristics of the sample at the 2012 data collection—unweighted (*N* = 7076).

​	M ± SD (range)	%
Demographic factors		
Age	67.0 ± 10.4 (50-100)	​
Gender (female)	​	54.6%
Race/ethnicity	​	​
Non-Hispanic white	​	69.1%
Non-Hispanic black	​	17.1%
Hispanic	​	10.5%
Non-Hispanic other	​	3.4%
Socioeconomic factors		
Education	​	​
High school diploma or below	​	63.5%
College degree or above	​	36.5%
Family income ($10,000)	7.9 ± 11.7 (0–144)	​
Health status		
Self-rated health (poor health)	​	12.2%
Number of chronic conditions	​	​
No condition	​	58.6%
One condition	​	30.8%
Two or more conditions	​	10.6%
Depression	0.8 ± 1.4 (0–8)	​
Childhood traumatic event		
No event	​	69.8%
One event	​	23.1%
Two or more events	​	7.2%
Adulthood traumatic event		
No event	​	45.7%
One event	​	29.7%
Two or more events	​	24.5%

**Figure 1.**
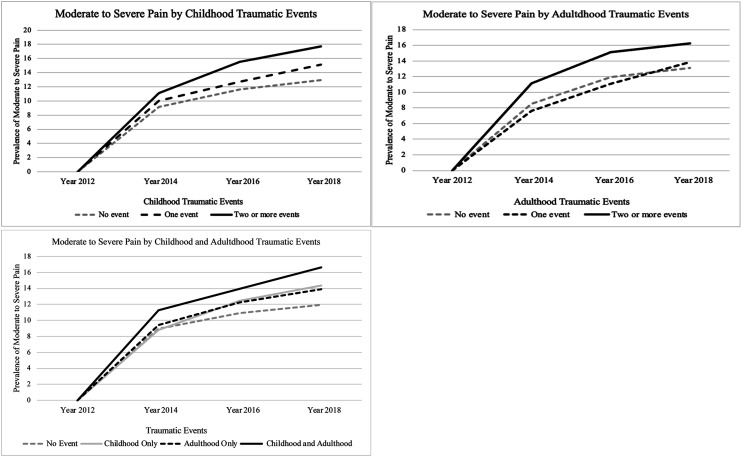
Prevalence of Pain by Childhood and Adulthood Traumatic Events. *Note.* Chi-square tests were conducted to compare the prevalence of pain each year by the number of adulthood traumatic events, and all comparisons were statistically significant at alpha 0.05.

Figure 1 shows the results of bivariate analysis between childhood and adulthood traumatic events and moderate-to-severe pain over time. In 2012, no respondents had reported pain, as the sample was restricted to those without moderate-to-vigorous pain at baseline. By 2018, pain prevalence increased across all groups, with higher rates among those with more traumatic events in childhood and adulthood. Specifically, for childhood traumatic events, the prevalence of pain among individuals with two or more traumatic events in childhood was 11% in 2014, 16% in 2016, and 18% in 2018, while those without childhood traumatic events had 9% in 2014, 12% in 2016, and 13% in 2018. A similar trend was observed for adulthood traumatic events. The prevalence of pain among individuals with two or more traumatic events in adulthood was 11% in 2014, 15% in 2016, and 16% in 2018. Furthermore, the gap between those with fewer and more traumatic events continued to widen over time. Lastly, the third panel shows the prevalence of pain across four groups: no event, childhood traumatic events only, adulthood traumatic events only, and both childhood and adulthood traumatic events. We observed the highest pain prevalence consistently among individuals who experienced both childhood and adulthood traumatic events.

As shown in Table 2, the results of multilevel logistic regression models showed the association between adulthood traumatic events and moderate-to-severe pain. In Model 1, after accounting for sociodemographic characteristics, individuals with two or more traumatic events in adulthood had higher odds of pain (OR = 1.58, 95% CI = 1.23, 2.03). In Model 2, after adjusting for physical and mental health problems, individuals reporting two or more adulthood traumatic events had higher odds of moderate-to-severe pain (OR = 1.34, 95% CI = 1.05, 1.71). When including both childhood and adulthood traumatic events in the model, we observed that adulthood traumatic events remained statistically significant. Specifically, experiences of two or more adulthood traumatic events were associated with 28% higher odds of moderate-to-severe pain (OR = 1.28, 95% CI = 1.00, 1.64). Experiences of two or more childhood traumatic events were also associated with 57% higher odds of pain (OR = 1.57, 95% CI = 1.11, 2.21).

**Table 2.** Multilevel logistic regression models of pain, 2014–2018.

Variables	Model 1		Model 2		Model 3	
	OR	95% CI	OR	95% CI	OR	95% CI
Age	1.00	0.99, 1.01	1.00	0.99, 1.01	1.00	0.99, 1.01
Female	1.35^*^	1.06, 1.71	1.42^**^	1.13, 1.78	1.48^**^	1.17, 1.86
Race/ethnicity (reference: Non-Hispanic white)						
Non-Hispanic black	1.11	0.87, 1.42	0.93	0.73, 1.19	0.92	0.72, 1.18
Hispanic	1.21	0.79, 1.85	1.01	0.68, 1.50	1.00	0.68, 1.47
Non-Hispanic other	1.70^*^	1.05, 1.74	1.38	0.85, 2.26	1.42	0.88, 2.28
Education (reference: high school diploma or below)	0.73^**^	0.58, 0.91	0.79^*^	0.64, 0.98	0.82	0.66, 1.01
Family income ($10,000)	1.00	0.99, 1.01	1.00	0.99, 1.01	1.00	0.99, 1.01
Self-rated poor health	​	​	1.92^***^	1.41, 2.60	1.92^***^	1.42, 2.60
Number of chronic conditions	​	​	1.24^**^	1.09, 1.42	1.24^**^	1.09, 1.41
Depression	​	​	1.17^***^	1.11, 1.23	1.17^***^	1.11, 1.23
Childhood traumatic events (reference: no event)						
One event	​	​	​	​	1.28	0.97, 1.70
Two or more events	​	​	​	​	1.57^*^	1.11, 2.21
Adulthood traumatic events (reference: no event)						
One event	1.10	0.81, 1.49	1.06	0.78, 1.43	1.04	0.77, 1.40
Two or more events	1.58^***^	1.23, 2.03	1.34^*^	1.05, 1.71	1.28^*^	1.00, 1.64
Time (years since 2012)	1.19^***^	1.13, 1.26	1.17^***^	1.11, 1.23	1.17^***^	1.11, 1.23

*Note*. *p < .05. **p < .01. ***p < .001.

## Discussion

The current study was conducted to explore the relationships between cumulative adulthood trauma exposure and pain outcomes over 6 years among middle-aged and older adults, after controlling for childhood trauma exposure. Our findings highlight the significant role of cumulative trauma across the life course in shaping pain experiences in older adulthood. While exposures to single adulthood traumatic events were not significantly associated with pain onset in comparison to those who did not experience any traumatic events in adulthood, middle-aged and older adults with a history of multiple trauma exposures in adulthood are more likely to report pain than those who did not experience a traumatic event, which partially supports our first hypothesis. Our findings also support our second hypothesis, as cumulative adulthood trauma was associated with greater risk of pain onset. This is congruent with the small amount of research conducted in this area on older adult populations specifically.^5^ It is also consistent with research demonstrating worse health outcomes are associated with more exposures to childhood trauma,^8,10,11^ and thereby suggests a similar dose-response relationship exists between cumulative adulthood exposures and pain. This relationship can be explained by physiological mechanisms, as repeated adulthood trauma exposure may affect the nervous system and stress-response systems, including activation of the hypothalamic-pituitary-adrenal (HPA) axis, which may lead to dysregulated pain processing and increase vulnerability to pain conditions in later life.^38^ Additionally, our findings were consistent with previous research studies demonstrating that a combination of trauma exposures in both childhood and adulthood, whether considered together or separately, was associated with more deleterious pain outcomes in adulthood compared to those with fewer or no trauma exposures.^12,13^

In support of our third hypothesis, we found that rates of pain prevalence were highest among those exposed to trauma in both childhood and adulthood, as opposed to those exposed to trauma during only one of these life stages. Nonetheless, our results indicated that both types of cumulative exposures were uniquely associated with pain onset among middle-aged and older adults. This is in contrast to the findings of Wang et al.,^14^ who observed relationships between traumatic exposures in adulthood, but not childhood, and late-life pain outcomes. This may be due to a focus in the previous studies on trauma variables specifically related to abuse and violence,^12,14^ as opposed to the current study, which examined a broader range of traumatic experiences although additional investigation is required to determine this with certainty. If true, this would provide additional support to the findings of previous studies demonstrating the importance of examining cumulative trauma over exposure type when studying the relationship between trauma and pain outcomes in later life.^11^

Across our results, the number of chronic health conditions, depression, and poor self-rating of health were all significantly associated with pain outcomes among middle-aged and older adults. Our findings were consistent with previous studies demonstrating similar relationships between poor self-rated health, physical and mental health, and pain,^18–20^ particularly depression.^22,23^ It is notable that in our results, cumulative trauma exposures in childhood and adulthood significantly contributed to adult pain outcomes, even after controlling for these variables.

Overall, traumatic experiences occurring during adulthood may have unique implications for pain risk, as they may interact with age-related vulnerability and accumulated health conditions,^39^ highlighting the importance of screening for adulthood trauma exposures, as cumulative exposures can increase the likelihood of experiencing late-life pain. Health care policies should reflect this by ensuring coverage for routine screenings and early implementation of trauma-informed care approaches that can help mitigate the potential for worsening pain over time.

## Limitations

Our findings must be considered in the context of several limitations. Several of the events included as part of the trauma exposure variables for children (e.g., repeating a year of school, parental alcohol, or drug use that caused family problems) and adults (e.g., having had a life-threatening illness or having had a spouse or child with a life-threatening illness or addiction to drugs or alcohol) would not be defined as “trauma” per the diagnostic criteria of American Psychiatric Association’s (APA) revised Fifth Edition of the Diagnostic and Statistical Manual (DSM-5-TR).^40^ According to the DSM-5-TR’s diagnostic Criterion A for PTSD,^40^ only events that include “exposure to actual or threatened death, serious injury, or sexual violence” (p. 301) through direct experience, or directly witnessing the events, learning that they happened to close others, or being repeatedly exposed in the workplace via the nature of one’s job (i.e., first responders) would be considered traumatic. However, this criterion has received critique for being too limiting,^41^ and some have used alternative definitions emphasizing the personal and subjective nature of traumatic experiences in its place.^42^ Additionally, some traumatic events included in our analysis (e.g., having fired a weapon in combat or been fired upon in combat, having been the victim of a serious physical attack or assault, or experiencing a life-threatening illness or accident), may result in physical injury or health conditions that contribute to pain. Thus, it is possible that some associations may reflect the direct physical consequences of these events. While we did not examine PTSD as an outcome in this study, similar findings have been reported with respect to pain outcomes. For example, results from a study conducted by Gasperi and colleagues^43^ revealed that Criterion-A-specific trauma exposures did not determine whether combat veterans experienced both pain and PTSD symptoms. Other researchers conducting previous studies in this area have also examined trauma exposure variables that fell outside of the scope of the DSM-5-TR’s definition of trauma for PTSD.^5,10,11^ Another limitation is that our study did not analyze some of the types of trauma exposures included in previous studies, such as sexual abuse and violence,^5,13^ as the HRS did not include information on all of these specific exposures. Nonetheless, results of prior studies indicate that the cumulative impact of trauma, also examined in this study, yielded a greater impact on adult pain outcomes than any specific trauma type. It is also important to consider that we did not differentiate between the different types of trauma exposures analyzed in our study. It is possible that different types of traumatic exposures may be associated with distinct pain outcomes. Future research examining these distinctions may provide nuanced insight into the relationships between trauma exposure and pain in later life. Also, our analytic sample was limited to those who were free of pain over 10 years prior to our analysis. This prevented us from examining whether traumatic experiences are associated with the exacerbation of existing pain conditions, thereby warranting future research to investigate both the development of pain and the worsening of pre-existing pain. Although the HRS 2020 has been released, we only used data up to the HRS 2018, due to the unique and potentially confounding impact of the COVID-19 pandemic on pain outcomes during that period. Finally, the HRS sample of middle-aged and older adults spanned a wide age range (50–100 years old). The long-term impacts of trauma, combined with the negative effects associated with the natural aging cycle, could be worse for the oldest adults. Our results demonstrated that pain outcomes worsened with time, regardless of age. However, a more specific analysis comparing different age categories of older adults might reveal group differences, and we encourage future studies of this nature.

## Conclusions

Our results provide insight into relationships between cumulative trauma exposures during childhood and adulthood and pain outcomes in middle-aged and older adults. In congruence with prior research, we found that cumulative trauma exposure during both childhood and adulthood is associated with a higher risk of pain onset. By using data from the nationally representative sample of middle-aged and older adults, our findings offer broader relevance to the US adult population. We extend existing literature by demonstrating that multiple adulthood trauma exposures uniquely contribute to middle-aged and older adults’ pain outcomes, even among those with a childhood trauma history. We encourage future researchers to bolster epidemiological findings by examining how the physiological consequences of trauma impact the natural aging process.

## Data Availability

The Health and Retirement Study (HRS) is a publicly available data sponsored by the National institute on Aging (NIA U01AG009740). The data are available on the HRS website at https://hrs.isr.umich.edu/data-products
